# Boosting k-means clustering with symbiotic organisms search for automatic clustering problems

**DOI:** 10.1371/journal.pone.0272861

**Published:** 2022-08-11

**Authors:** Abiodun M. Ikotun, Absalom E. Ezugwu

**Affiliations:** 1 School of Mathematics, Statistics, and Computer Science, University of KwaZulu-Natal, Pietermaritzburg, KwaZulu-Natal, South Africa; 2 Department of Computer Technology, Yaba College of Technology, Lagos, Lagos State, Nigeria; National Taiwan University of Science and Technology, TAIWAN

## Abstract

Kmeans clustering algorithm is an iterative unsupervised learning algorithm that tries to partition the given dataset into *k* pre-defined distinct non-overlapping clusters where each data point belongs to only one group. However, its performance is affected by its sensitivity to the initial cluster centroids with the possibility of convergence into local optimum and specification of cluster number as the input parameter. Recently, the hybridization of metaheuristics algorithms with the K-Means algorithm has been explored to address these problems and effectively improve the algorithm’s performance. Nonetheless, most metaheuristics algorithms require rigorous parameter tunning to achieve an optimum result. This paper proposes a hybrid clustering method that combines the well-known symbiotic organisms search algorithm with K-Means using the SOS as a global search metaheuristic for generating the optimum initial cluster centroids for the K-Means. The SOS algorithm is more of a parameter-free metaheuristic with excellent search quality that only requires initialising a single control parameter. The performance of the proposed algorithm is investigated by comparing it with the classical SOS, classical K-means and other existing hybrids clustering algorithms on eleven (11) UCI Machine Learning Repository datasets and one artificial dataset. The results from the extensive computational experimentation show improved performance of the hybrid SOSK-Means for solving automatic clustering compared to the standard K-Means, symbiotic organisms search clustering methods and other hybrid clustering approaches.

## 1. Introduction

Cluster analysis is an aspect of data analysis where data objects are distinctly grouped into clusters. The data objects within the same cluster share more intrinsic characteristics and differ from data objects in other clusters [1]. The primary objective of data clustering is to minimize the intra-cluster distances while maximizing the inter-cluster distances. Smaller intra-cluster distances imply more robust and compact clusters [2]. Clustering algorithms have been widely applied in solving many problems in various fields, including pattern recognition [3], mathematical programming [4], data mining [5], social network analysis [6], image analysis [7], market research [8], customer segmentation [9], machine learning [10], data analysis [11] and data summarization [12].

Clustering algorithms are usually categorized as either hierarchical clustering or partitional clustering. The hierarchical clustering algorithms iteratively generate clusters in a top-down or bottom-up hierarchical format to produce a dendrogram structure. The dendrogram reflects the hierarchical relationship of the formulated clusters, which results from exploring the data objects on different levels of granularity during the clustering process [13]. Data objects are merged (bottom-up approach) or splitted (top-down approach) based on the similarity or dissimilarity among the data objects through linkage metrics. The hierarchical clustering algorithms can handle datasets with any objects of any attribute datatype because of their ability to handle any similarity measure and their exploration capability at a flexible level of granularity. However, once an object has been assigned, it can not be reassigned during hierarchical clustering, implying that wrongly assigned data objects can not be corrected. Hierarchical clustering also suffers from high computational complexity [14], making it unsuitable for large-scale clustering datasets. Berkin, Beche, and Randall [15] noted the problem with identifying specific termination criteria for hierarchical clustering methods and its sensitivity to noise and outliers, making it less robust in handling cluster analysis. Moreover, the generation of the hierarchical structure adds the burden of higher memory requirements which, according to Fraley and Raftery [16] is proportional square of the number of initial clusters generated during the clustering process.

The partitional clustering algorithms use an optimization criterion to partition a given dataset into a set of disjoint clusters [13]. Data objects are clustered to recover the naturally existing groups within a given dataset. The partitioning clustering methods offer a better alternative for clustering large datasets to avoid the construction of a dendrogram, which in the case of large datasets is generally considered computationally prohibitive [17]. The partitional clustering algorithms commonly use the squared error criterion as their optimization function to find the partition for which a fixed number of clusters minimizes the square error [13]. Data objects are iteratively assigned to an initial dataset partition such that the assignment reduces the square error. According to Nagpal [18], having a good initial partition highly enhances the clustering solution. Selection of well-distanced data objects among the existing data objects usually turned out to be good seed points for partitional clustering algorithms. Moreover, the number of clusters is indirectly proportional to the square errors. That is, the larger the number of clusters, the lesser the square errors. However, minimization is only guaranteed for a fixed number of clusters.

The square error criterion is also observed to produce compact and well-separated clusters and is less computationally demanding compared with other criterion functions [19]. However, using square error criterial can result in local minimal convergerce resulting in inconsistent cluster output. This implies that different cluster outputs are obtained from different initial partitions, especially if initial data seeds are close [18,20]. According to Sanse and Sharma [21], obtaining a global optimal clustering solution using partitional clustering can not be guaranteed. The partitional clustering algorithm is considered an NP-hard optimization algorithm whenever k>3 [22,23]. As such, a typical partitional clustering algorithm requires several runs with different starting partitions from among which the best cluster output is selected as the optimal [17]. According to Suganya et al. [24] and Jain and Dubes [19], the requirement of initial specification of the number of clusters is a major disadvantage of the partitional clustering algorithm. It leads to the arbitrary choice of cluster number for datasets whose number of inherent clusters is not known apriori, resulting in wrong clustering output. Moreover, partitional clustering algorithms assign data objects to the closest centroid to generate clusters whose sizes are approximately similar in sizes justifying their biasness towards spherically shaped clusters and their inability to effectively handle high-dimensional datasets and those whose clusters are highly connected [25].

In recent times, nature-inspired metaheuristics have been adopted to find solutions to complex challenges in cluster analysis that cannot be resolved using the traditional clustering algorithms [2,22,26]. They automatically identify and classifies unlabelled data points in real-world data and have been found to be more flexible in handling clustering problems across different fields. These automatic clustering algorithms spontaneously determine the optimum number of clusters in a dataset with their corresponding cluster structures, thus removing the need to specify the number of clusters apriori [13]. Automatic clustering automatically finds the most suitable number of clusters in a dataset and, at the same time, divides the data objects into appropriate clusters [27]. They are mostly adopted in large-scale and high-dimensional real-life datasets where the number of clusters is not known. They treat clustering problems as optimization problems to minimize the dissimilarity between clsuters, minimizing intra-cluster distance while maximizing the inter-cluster distance. According to Ezugwu et al. [26], they are designed to effectively and efficiently handle complex and high-dimensional real-world problems. They are characterised by higher heuristic search and can search for the most promising optimal solution. They balance intensification search with diversification search during the search process. They exhibit a higher possibility of finding global optimal solutions to clustering problems. Their performance in automatic clustering is superior to the traditional clustering algorithms when considering the convergence speed and clustering solution quality.

Some of the existing nature-inspired algorithms that have been employed in literature to solve automatic clustering problems include Artificial Bee Colony [28], Differential Evolution Algorithm [29], Bacterial Evolutionary Algorithm [30], Firefly Algorithm [31], Genetic Algorithm [32], Particle Swarm Optimization Algorithm [33], Invasive weed Optimization Algorithm [34], Ant Colony Optimization, [35] and Symbiotic Organism Search Algorithm [36]. In recent times, newer meta-heuristic algorithms have been proposed, such as Monarch Butterfly optimization [37], Moth search algorithm [38], Slime Mould algorithm [39], Dwarf Mongoose algorithm [40], Hunger Games Search [41], Harris Hawks Optimization [42] and Colony Predation Algorithm [43]. In spite of the effectiveness of the nature-inspired metaheuristic algorithms in finding the solution for automatic clustering problems, individual algorithm exhibit the limitations peculiar to their algorithmic structure affecting their overall performance. In order to improve their performances for automatic clustering, two or more of these algorithms are combined to build an hybridized algorithm that harnesses their mutual benefits for effective and efficient cluster output. Based on this context, a hybridisation of the mostly used partitional clustering algorithm- K-means -with Symbiotic metaheuristic algorithm-a nature inspired metaheuristic algorithm is proposed in this paper.

The K-means algorithm [44] is among the top ten most used partitional clustering algorithms in data analysis [16]. It uses an optimization criterion that minimizes the distance between the data objects within a cluster and their cluster centres while maximizing the distance between the centre of each cluster [45]. K-means clustering algorithm has been widely accepted due to its implementation simplicity, the high computational speed with a linear time complexity (*O(n)* time where n is the number of records in the dataset) as well as its ability to identify high-density regions based on the set of cluster centres and radii which provides insights into the data [16].

Despite these qualities, K-means exhibits some drawbacks common to the partitional algorithm mentioned earlier based on its algorithmic structure [46] and its inductive least sum of squares principles [47]. The need to specify k apriori as an input parameter is one of the problems of the algorithm. For big data and high-dimensional datasets, guessing the right number of clusters in advance can be an extremely challenging task for a user [48]. Two, the K-means algorithm is susceptible to the initial cluster centres. The initial cluster centroids are selected randomly in the classical K-means algorithm, and poor cluster seeds will produce poor clustering results. Third, the K-means algorithm adopts simple hill-climbing techniques to optimize its objective function, which is susceptible to getting trapped into the local optimum. Moreover, the presence of noise and outliers affects the performance of K-means because it assumes all clusters have a similar spread and equal density. This assumption misguides the algorithm when updating the cluster centres. The aim of this paper is to boost the performance of K-means and extend it to make it suitable for handling automatic clustering algorithm.

The Symbiotic Organism Search [49] is a metaheuristic algorithm that simulates the technique of symbiotic interactions among organisms in an ecosystem for their survival. It was proposed by Cheng and Prayogo [49] as a simple but powerful metaheuristic algorithm that adopts the search strategy of a population-based algorithm in searching for the optimum solution in the solution space. It iteratively searches for a global optimum solution using a population of candidate solutions through optimization of a given objective function. SOS has been reported to have a better searching quality and searching efficiency when compared with other metaheuristic algorithms such as GWO, which was noted as being superior to other existing metaheuristic algorithms in several reported comparisons [50,51]. The SOS algorithm is credited with performance stability because it does not use tunning parameters. It has the main advantage over most other metaheuristic algorithms in that it requires no specific algorithm parameters [49] and is easier to implement. This paper proposes a new hybrid clustering algorithm called SOSK-means, which combines the SOS algorithm with the classical K-means algorithm for automatic clustering. The traditional K-means clustering algorithm has been hybridized with some of the existing nature-inspired metaheuristic algorithms [51]. However, the focus in many of these hybridized algorithms is on enhancing the capability of the respective nature-inspired algorithm in handling automatic clustering problems. This study aims to boost K-means performance by hybridizing it with SOS to resolve the problems of specifying cluster numbers as input parameters and the selection of cluster centroids randomly, thereby avoiding the possibility of local minimal convergence.

The SOS algorithm has been chosen based on its numerous capabilities, as highlighted in [52] and [53]. The SOS algorithm requires only the basic parameter that is needed for optimization operation, such as the population size, the problem dimension and the maximum number of iterations. Other population-based metaheuristics such as GA, PSO, DE, and FA require additional parameters that need adjustment or fine-tuning for optimal performance [52]. According to Abdullahi and Ngadi [54], traditional metaheuristic algorithms like GA, PSO, and ACo suffer from entrapment in local minima, slow convergence and high computational complexity. The mutualism and commensalism phases of the SOS algorithm offer an excellent exploitation capability to the algorithm. The best solution is used as the reference point while exploiting the search space for a better solution in the neighbourhood of the current best solution. In the SOS algorithm, inferior solutions are eliminated through the cloning and mutation operation of the parasitism phase. According to [52], only a few algorithms exhibits all these characteristic.

The searching quality of the SOS algorithm has been confirmed to be superior to other high-performing metaheuristic algorithms [50,53]. SOS was compared with several metaheuristic methods by Pierezan and Coelho [50], and in almost all the problems used for testing the algorithms, SOS was found to perform better in its searching quality. This performance was further justified by the work of Chauhan and Kotecha [55], where the SOS algorithm was compared with GWO in the petrochemical industry’s production planning problem. The SOS algorithm had a better performance in all the eight testing cases. Moreover, from the literature, hybridizing K-means with SOS is still relatively new. The K-means algorithm’s performance can be substantially enhanced in solving clustering problems if SOS with all the identified qualities can be adopted for the hybridization. This is the motivation of this research work.

The Davies-Bouldin (DB) [56] and Compact-Separated (CS) [57] validity indices are employed in the hybridized algorithm for the determination of the optimal number of clusters. The two CVIs are selected because they share the same rationale with the primary purpose of clustering in their cluster validity measurement approach. They seek well-separated clusters with the maximum score for the between-cluster separation and more compact clusters with within-cluster scatter at the minimum [57]. Moreover, the CS Index works well in identifying clusters with different sizes and densities. The DB Index is among the top-performing CVIs in some reported CVI comparison literature [58,59]. It is useful in guiding cluster seeking algorithms independently of the number of clusters and the partitioning approach used for the clustering [56]. Eleven benchmark UCI datasets with one artificial dataset were used to validate the algorithm’s performance.

The remaining section of the paper is organized as follows: a review of literature on hybrid K-means for automatic clustering is presented in section 2, while the problem statement and the methodology of the proposed hybrid Swarm organism Search Algorithm with K-means together with its implementation are described in section 3. The experimental setting of the study, the datasets characteristic, the parameter configurations, and the simulation experimentation results obtained from the proposed SOSK-means are discussed in Section 4. The conclusions and the future research directions are presented in section 5.

## 2. Literature review

In the bid to enhance the performance of the K-means algorithm, several proposals on K-means variants have been made and implemented in literature to address the various problems associated with the classical algorithm [46]. Hybridizing K-means with nature-inspired metaheuristic algorithms is one of the research methods adopted to resolve the initialization problems and the local minimal convergence issues common with K-means. Only a few of these hybridized algorithms involving K-means focused on solving problems in automatic data clustering. Some of the metaheuristic-based hybrid algorithms that have been proposed for handling automatic data clustering analysis and their performances can be found in the work of Agbaje, Ezugwu and Els [2]. However, the emphasis here is on those involving the basic K-means algorithm. Mustafi and Sahoo [60] proposed a hybrid approach that combined genetic algorithms with differential evolution algorithms to enhance the K-means algorithm’s initialisation process. The primary aim of their proposed algorithm was to improve the initial choice of the K-means algorithm’s cluster centres and generate the required number of clusters. The genetic algorithm framework was explored for obtaining the original seed points for the K-means, while the differential evolution heuristic was used to generate the required number of clusters. Their result was compared with the basic implementation of the K-means algorithm using standard parameters. It showed a significant reduction in the possibility of convergence to the local optimal by the K-means algorithm.

In Sinha and Jana [61], the genetic algorithm and k-means clustering algorithm were combined in a two-phased hybridization approach using Mahalanobis distance as a fitness function for K-means initial cluster center generation. The first phase used a genetic algorithm with Mahalanobis distance to better represent the initial data. The intermediate output from phase one serves as input for the K-means algorithm, which uses the K-means++ initialization technique to produce the final output in phase two. Islam et al. [62] reported an advancement in genetic algorithm-based clustering where the searching approach of the genetics was combined with the fast K-means hill-climbing cycles for fast generation of the high-quality clustering result. Their algorithm addressed the problems of prior specification of k and selection of initial cluster centers randomly. K-means with different clusters are applied several times to explore the data and rank the chromosomes based on their fitness. The k value with good clusters with the highest rank fitting chromosomes is selected as the initial population.

Zhang and Zhou [63] presented another genetic algorithm-based clustering coupled with K-means++. They used a canopy and K-mean++ for initial population generation and initial seedlings, respectively, without specifying the number of clusters apriori. They adopted a sharing-based niche that maintains population diversity to capture global optimal. They used adaptive crossover and mutation probabilities to avoid local optimum convergence. Their main aim is to improve the performance of genetic algorithm-based clustering techniques. Kapil, Chawla and Ansari [64] used a genetic algorithm to optimized K-means to override the K-means initialization problem. Each data object acts as a candidate for cluster centroid with the range of the data set represented as the chromosome. The genetic algorithm is applied to generate the fittest instance, which is then selected as the cluster centroids for the k-means clustering. Their result showed correctly clustered instances with a reduced sum of squared errors compared with the traditional k-means clustering. In Rahman and Islam [65], a genetic algorithm-based clustering is proposed which finds accurate cluster numbers automatically with cluster centres that are of high quality. The high-quality cluster centres from the genetic algorithm-based clustering then serve as initial centroids for the K-means to produce high-quality clustering results.

Xiao et al. [66] hybridized a quantum-inspired genetic algorithm with K-means automatic clustering. The typical genetic algorithm operations of Q-bits were used in conjunction with Q-bit representation for exploration and exploitation in discrete 0–1 hyperspace using rotation operation of the quantum gate. Their algorithm found the optimal number of clusters and provided optimal cluster centroids. As with other genetic algorithm-based hybridized clustering algorithms, parameter tunning plays a significant role in the performance of their algorithm. For instance, to find the optimal solution, the maximal iteration number must be as substantial as possible, which invariably impairs the performance in terms of execution time. In all of these hybridization approaches involving genetic algorithms and K-means for clustering, there is a common problem of parameters tunning for an optimum clustering solution. Also, the increased complexity reduced the performance of the hybrid clustering algorithm when dealing with large-scale and high-dimensional data. Moreover, high-quality clustering resulted in higher computational time. Kuo, Suryani and Yasid [67] proposed a hybrid algorithm for automatic clustering that combines K-means with a differential evolution algorithm. Their proposed algorithm, whose primary aim was to improve the performance of the DE algorithm, requires no specification of the cluster number apriori. The DE algorithm generates the initial cluster centers for the K-means algorithm, while the K-means algorithm was then employed to fine-tune the cluster centres for better clustering results.

Silva et al. [68] applied a U-control chart on automatic clustering differential evolution (ACDE) to determine the number of K-means clusters. Their work automated the determination of the k activation threshold in ACDE and the resulting number of clusters fed as input to the K-means algorithm. Their result showed improved clustering performance. Cai et al. [69] also presented a clustering-based DE hybridized with one-step K-means clustering for a more effective and efficient DE. The one-step K-means efficiently utilize the population information by acting as a large numbered multi-parent crossover operator. It enhances the hybrid DE in balancing the evolutionary’s exploration and exploitation process. The number of clusters is generated as a random integer between the two and the square root of the population size. Their approach showed an enhancement in the performance of DE in terms of the final result’s quality and reduction in the number of evaluation fo fitness function. The main purpose of hybridizing with K-means is to improve the exploitation capability of DE. However, the hybridized algorithm’s performance is sensitive to the population size. The probability of finding the correct search direction decreases as population size increases.

In Cobos et al. [70], the global-best harmony search is hybridized with the K-means for automatically clustering web documents. The algorithm used the Bayesian information criterion to determine the number of clusters automatically. The Global-best harmony search was used as the global search strategy in the entire solution space, while the K-means algorithm was used as a local strategy to improve the clustering solutions. Their result showed a better precision than the singular algorithm, but the initialization parameters profoundly affect the algorithm’s performance. There is a need for proper tunning of the input parameters to achieve an optimum result. Kuo and Zulvia [27] also proposed an automatic clustering algorithm based on an improved artificial bee colony optimization algorithm with the K-means algorithm. The onlooker bee exploration scheme was improved when their movement was directed to a better location in their algorithm. The improved ABC provides a better initial cluster centroid for the K-means algorithm presenting the data centre location as a better location for the onlooker bee to accelerate the exploitation step. To avoid local optimal convergence of the onlooker bees as they move towards the data centre, the movement length of each onlooker bee is considered, which is affected by the random number generated for the onlooker bee. Their work included a parameter analysis to find the best parameter setting for the optimal performance of their proposed algorithm. Their result showed improved performance regarding the computation time required for conducting the scout bee phase and better food generation by the onlooker bees. Other metaheuristics-based K-means hybridized algorithms for automatic clustering reported in the literature can be found in [71–73]. As is the case with basic metaheuristic algorithms, basic issues involving the need for parameter tuning, increased computational complexity, and higher computational time for achieving better quality clustering results are prominent in most of the hybridized metaheuristic algorithms with K-means.

The Symbiotic Organism Search [49] is a nature-inspired metaheuristic algorithm that simulates the technique of symbiotic interactions among organisms in an ecosystem for their survival. SOS was originally designed by [49] to solve optimization problems in continuous solution space. It was initially used to solve four structural engineering design problems and 26 unconstrained mathematical problems to establish the performance of the algorithm. However, SOS has been adopted and transformed to apply in other problem spaces. A discrete version of the SOS algorithm was introduced by [74], using it to solve multiple project scheduling problems. In Discrete SOS (DSOS), the continuous solutions are transformed into discrete solutions. It incorporates a function that converts the real-value variables into integer values constrained within the feasible solution space. Further DSOS research can be found in Ezugwu and Adewumi [75] and Sharma and Verma [76].

Three modified versions of the classical SOS were proposed by Tejani, Savsan and Patel [77] to solve structural design optimization problems. They incorporated new adaptive benefit factors which were combined with the standard SOS benefit factors to achieve a good balance between exploitation and exploration for performance efficiency improvement. Their adaptive SOS performed better than the classical SOS. Nama et al. [78] proposed an improved SOS called I-SOS, where a random weighted reflective parameter was introduced to the classical SOS along with an extra predative phase for performance enhancement. The random weighted reflective parameter forms new sets of mutualism and commensalism update phases in the ecosystem. In the predative phase, the predator harms its relating partner and probably kills it. The predation vector replaces the worst organisms in the population. The improved version of the SOS algorithm reported better performance than other methods in finding a solution to the Optimal capacity of Gas production facility and Gas transmission compressor design problem. Other variants to the standard SOS reported in literature includes Saha and Murkherjee [79], Chakraborty, Nama and Saha [80], Al-Sharhan and Omran [81], Nama, Saha and Sharma [82]. The SOS algorithm has also been modified to solve multiobjective optimization problems where more than one objective function is required to be simultaneously optimized. Research reports covering this aspect can be found in Cheng and Prayogo [74], Tran, Chen and Prayogo [83], Panda and Pani [84], and Ayala et al. [85].

Hybridization of the SOS algorithm with other metaheuristic algorithms is another area that has been explored in the literature to improve the performance of the classical SOS algorithms. The literature has established that combining multiple algorithms as a hybrid produces better and more robust solutions than the sole use of the individual algorithm [86,87]. Several hybridized algorithms involving SOS have been reported in the literature. Abdulahi and Ngadi [88] proposed a hybrid algorithm combining SOS and SA (Simulated Annealing) to optimize the task scheduling process in a cloud computing environment. A hybrid of SOS called HSOS was proposed by Nama et al. [89], combining the standard SOS with Simple Quadratic Interpolation(SQI) to leverage the exploitation capability of SOS with the exploration potential of the SQI. HSOS recorded a high searching capability to achieve a global optimum. Ezugwu et al. [87] applied the SOSSA in solving the travelling salesman problem (TSP). The performance of the hybridized algorithm was evaluated using different TSP benchmark sets from the TSPLIB, recording a low convergence rate with near-optimal solutions in most cases and near-optimal solutions in some cases.

SOS algorithm has also been used for solving automatic clustering problems in literature. However, only a few involve SOS’s hybridisation with other metaheuristic algorithms for automatic clustering. Zhou et al. [36] proposed automatic data clustering using the nature-inspired symbiotic organism search algorithm. The SOS algorithm was used to solve clustering problems on ten standard datasets from the UCI machine learning repository. The clustering performance of the algorithm was compared with six other metaheuristic-based clustering algorithms–Particle Swarm Optimization, Differential Evolution, Flower pollination, Cuckoo Search, Artificial Bee Colony, Multi-verse Optimizer and K-means. The means and standard deviations were used as comparison measures for the optimal performance of SOS with these other algorithms. The experimental result showed that SOS outperformed all the algorithms compared to its convergence speed and solution quality. It also demonstrated a superior level of stability. Zhou et al. [36] work is similar to this research work because SOS was used in solving automatic clustering algorithms. The use of the SOS algorithm alone limits the final clustering performance in terms of the solution quality compared with results obtained from hybridized algorithms such as our work in solving automatic clustering. As a recommendation for future research, they suggested the hybridization of the SOS algorithm with other algorithms to combine the advantages of the participating algorithms for better clustering solutions.

Yang & Sutrisno [53] integrated the automatic k-means clustering method with SOS to create subpopulations on the SOS initial solutions in their proposed clustering-based SOS algorithm called CSOS for high dimensional optimization problems. They aimed to enhance the SOS algorithm’s searching quality and searching efficiency by combining the concept of local and global searching through clustering. CSOS adopted an automatic cluster generation and merging method by dividing the ecosystem into several sub-ecosystems to create a faster algorithm using the automatic k-means algorithm. The k value for the k-means is determined as half of the ecosize to ensure that there are at least two solutions in every cluster. Clusters with only one solution are merged with the closest cluster at the initialization stage. Using twenty-eight benchmark performance and efficiency parameters, the CSOS algorithm was compared with GA, CRPSO, SaNSDE, rCMA-ES, GWO and SOS. It was also compared with other clustering-based metaheuristics—HSGA, ACVPSO, CDE and 2-MPCs-CDE using eight problems based on FE, SR, and best-found solutions. Their result showed improved performance compared with SOS in terms of computational speed in high-dimensional problems. Their work is different from ours in that K-means was introduced to improve the initialisation procedure of the SOS algorithm and can not be taken as a hybridization of the two algorithms. Their aim, as stated earlier, was to improve the quality and efficiency of the search process of SOS. Our focus is on enhancing the widely used K-means algorithm’s clustering performance and extending it to handle automatic clustering.

Rajah and Ezugwu [1] proposed and implemented four SOS-based hybrid algorithms–SOSFA, SOSDE, SOSTLBO, and SOSPSO for automatic partitioning of datasets without prior knowledge of the number of clusters in the datasets. Their main goal was to improve the overall performance of the basic SOS algorithm using a hybridization approach for automatic clustering. The proposed algorithms were evaluated using the Davies-Boulding clustering validity index based on the solution quality obtained. In their implementation, the SOS with its various hybridized algorithms were used to solve automatic clustering analysis problems using twelve UCI datasets. The performances of this hybridized algorithm were compared with SOS and some other state-of-the-art hybridized algorithms. Their result established that hybrid algorithms are powerful optimizations for solving real-life applications such as cluster analysis problems. Their result also revealed that the three hybrid algorithms (SOSFA, SOSTLBO, SOSPSO) outperformed the basic SOS algorithm while SOSDE performance was at par with the basic SOS algorithm. This work is also similar to ours because it focuses on hybridizing SOS with other metaheuristic algorithms and comparing their clustering performances using only the DB cluster validity index. In addition, our work exploits a hybridization of SOS with the traditional K-means clustering algorithm. A summary of the various hybrid algorithms involving either the K-means algorithm or Symbiotic Organism Search with other metaheuristic algorithms or both of them is given in Table 1.

**Table 1:** Summary of literature review on K-means hybridization with metaheuristic algorithms.

S/N	Algorithm	Reference	Method	Findings	Limitations
**1**	GA- and DE-based Heuristics hybrid algorithm	Mustafi and Sahoo [60]	Hybridised GA and DE with K-means	Improved initial seeding for the K-means algorithm with the requisite number of clusters	Proper tunning of basic GA input parameters is required with increased computational time and complexity
**2**	MapReduce-based hybrid algorithm	Sinha and Jana [61],	Hybridised GA with K-means using Mahalanobis distance as fitness function and K-means ++ initialization process	MapReduce-based K-means hybridized with GA for clustering in a distributed environment	Proper tunning of basic GA input parameters are required
**3**	GENCLUST++	Islam et al. [62]	Hybridised GA with K-means	Advancement in genetic algorithm-based clustering for quality clustering solutions with O(n) complexity	Proper tunning of basic GA input parameters is required with increased computational complexity
**4**	NCLUST	Zhang and Zhou [63]	Hybridised GA with K-means++	Genetic Algorithm-based hybrid clustering with maintained population diversity	Proper tuning of basic GA input parameters and increased computational time is required for a high-quality result.
**5**	Genetic K-means	Kapil, Chawla and Ansari [64]	Hybridised GA with K-means	Optimized K-means using GA for better K-means initialisation process using sample dataset as chromosomes	Proper tunning of basic GA input parameters is required with increased computational complexity
**6**	GENCLUST	Rahman and Islam [65]	Hybridised GA with K-means	Genetic algorithm-based clustering with automatic generated accurate cluster numbers and high-quality cluster centres	Proper tunning of basic GA input parameters is required with increased computational complexity
**7**	KMQGA	Xiao et al. [66]	Hybridised GA with K-means	Generation of an optimal number of clusters and optimal cluster centroids using Q-bits operations and representation	Proper tunning of basic GA input parameters is required, and increased computational complexity
**8**	ACDE-K-MEANS	Kuo, Suryani and Yasid [67]	Hybridised Improved DE with K-means	Automatic generation of a number of clusters	Proper tunning of basic DE input parameters is required
**9**	ACDE	Silva et al. [68]	Hybridised DE with K-means	Automatic generation of a number of clusters using U-control chart-based DE	Proper tunning of basic DE input parameters is required
**10**	CDE	Cai et al. [69]	Use of one-step K-means with DE	Improved performance for DE-based clustering	Proper tunning of basic DE input parameters is required. Higher computational time for better quality clustering
**11**	IGBHSK	Cobos et al. [70]	Hybridised Global best HS with K-meanS	Automatic clustering using BIC for determining cluster numbers for document clustering	Proper tunning of basic HS input parameters is required
**12**	iABC	Kuo and Zulvia [27]	Hybridised improved ABC with K-means	Better initial cluster centroid for the K-means algorithm with better and more stable clustering result	Required parameter analysis to achieve optimal performance. The higher computational time for better quality clustering
**13**	Classical SOS	Zhou et al. [36]	Using SOS Algorithm for solving clustering problem	Automatic clustering using classical SOS	Required parameter analysis to achieve optimal performance. Limited in performance as a single algorithm
**14**	CSOS	Yang & Sutrisno [53]	Integrate K-means with SOS	Uses K-means for the classical SOS algorithm’s initialization improvement to improve the searching quality and searching efficiency	The focus is on improving the classical SOS algorithm
**15**	SOSFA, SOSDE, SOSPSO, SOSTLBO	Rajah and Ezugwu [1]	Hybridized SOS with FA, DE, PSO and TLBO	Improving the performance of the basic SOS algorithms through hybridization	Proper tunning of basic participating metaheuristics input parameters is required

Automatic clustering using hybridization of nature inspire algorithms is still a new research area. Efforts are tailored toward reducing the computational complexity, parameter tunning and computational time to achieve optimal clustering results. This paper focuses on hybridizing SOS with K-means to achieve a better clustering performance while extending K-means advantage in solving automatic clustering problems. The strengths of the SOS algorithm of parameter-free characteristics with its excellent global search capability will be harnessed to automatically determine the number of clusters in the datasets and generate corresponding initial cluster centers for the classical K-means algorithm. The Davies Boulding Cluster Validity Index and the Compact-Separated (CS) index will be used to validate the clustering performance of the proposed algorithm.

## 3. Methodology

This section presents the computational model of the proposed SOSK-means algorithm. SOSK-means combined SOS algorithm with standard K-means algorithm to solve the automatic data clustering problem. An overview of the SOS algorithm is presented, describing the three phases of generating new solutions. This study solves the automatic data clustering problem using a hybrid algorithm combining the Symbiotic Organism Search algorithm and K-means algorithm. The proposed SOSK-means algorithm is implemented using the approach described in [53], which handles similar hybridizations.

### 3.1. Symbiotic organism search algorithm

The SOS algorithm is a nature-inspired metaheuristic algorithm that simulates the symbiotic interaction strategies of organisms for survival and propagation in the ecosystem [49]. As is common with other metaheuristic algorithms, SOS uses random variables and does not require substantial gradient information. However, unlike other metaheuristic algorithms, the operation of the SOS algorithm requires no specific algorithm parameters except for the general parameters common to population-based algorithms, such as the maximum number of iterations, population size and problem dimension. This exonerates the SOS algorithm from the problem of parameter tuning for an optimal solution. The SOS algorithm mimics the symbiotic interactions between a paired organism relationship to search for the optimal global solution in a continuous search space. In seeking the optimal global solution, the SOS iteratively employed a population of candidate solutions to the promising areas in the search space. It starts with an initial population which is referred to as the ecosystem.

The initial ecosystem comprises a group of organisms randomly generated for the search space, with each organism representing a candidate solution to the automatic clustering problem. A fitness value reflecting the degree of adaptation of the organism to the desired objective (automatic clustering) is associated with each organism. The SOS algorithm employed the three phases of symbiotic relationship: mutualism, commensalism, and parasitism, as a succession of operations to solutions in each iteration to generate new solutions for the next iteration. The main principle of each phase is based on the corresponding symbiotic relationship. In the mutualism phase, the two organisms involved benefit from the interaction. In contrast, in the commensalism phase, only one of the organisms receives benefit and the other neither benefit nor lose from the interaction. However, in the parasitism phase, one organism benefits while actively harming the other organism. The three phases are repeated at each iteration until the termination criteria are met.

#### Mutualism phase

A mutual relationship is defined between an organism *X*_*i*_ and another randomly selected *X*_*j*_ such that the association between the two organisms enhances their mutual survival rate within the ecosystem. *X*_*i*_ and *X*_*j*_ correspond to the i^th^ and j^th^ members of the ecosystem. Based on the mutualistic relationship between them, new solutions for X_i_ and X_j_ are generated using Eqs (1) and (2), respectively:

$$X_{inew}=X_{i}+rand(0,1)\times (X_{best}-X_{mutual}\times {BF}_{1})$$ (1)

$$X_{jnew}=X_{j}+rand(0,1)\times (X_{best}-X_{mutual}\times {BF}_{2})$$ (2)

$$X_{mutual}=\frac{X_{i}+X_{j}}{2}$$ (3)

*X*_*inew*_ and *X*_*jnew*_ represents the new solutions for the interacting organisms *X*_*i*_ and *X*_*j*j,_ respectively. The *X*_*best*_ represent the highest degree of adaptation, and it is modelled as the target point for the fitness increment of both organisms. The *X*_*mutual*_ expresses the characteristic relationship between the interacting organisms while the (*X*_*best*_−*X*_*mutual*_×*BF*_1_) reflects the mutualistic effort required to achieve the goal of increasing their survival in the ecosystem. The *BF*_1_ and *BF*_2_ are called the benefit factors, representing the level of benefit to each interacting organism. The values are randomly determined as either 1 or 2 to reflect if an organism has a partial or full benefit from the relationship. Interacting organisms’ values are updated if and only if their new fitness is better than their pre-interaction fitness, as reflected in Eqs (4) and (5), respectively.


$$X_{inew}=X_{i}+rand(0,1)\times (X_{best}-X_{mutual}\times {BF}_{1})$$

$$if\;f(X_{inew})>f(X_{i})$$ (4)

$$X_{jnew}=X_{j}+rand(0,1)\times (X_{best}-X_{mutual}\times {BF}_{2})$$

$$if\;f(X_{jnew})>f(X_{j})$$ (5)

#### Commensalism Phase

In the commensalism phase, the organism *X*_*j*_ is randomly selected to interact with the organism *X*_*i*_ as is the case in the previous phase, however, only *X*_*i*_ benefits from the relationship while *X*_*j*_ neither benefit nor suffer from the interaction. In this case, only organism *X*_*i*_ has a new solution which is generated using Eq (6) based on the commensal relationship between the interacting organisms.


$$X_{inew}=X_{i}+rand(-1,1)\times (X_{best}-X_{j})\;if\;f(X_{inew})>f(X_{i})$$ (6)

The (*X*_*best*_−*X*_*j*_) reflects the beneficial advantage X_i_ gets from interacting with X_j_ to increase its survival advantage in the ecosystem with respect to the highest degree in the current organism. Just as it was in the previous case, *X*_*i*_ is updated if and only if the new fitness is better than its pre-interaction fitness.

#### Parasitism phase

In the parasitism phase, the organism *X*_*i*_ is duplicated to create a parasite vector and then used a random number to modify the randomly selected dimensions. A host to the parasite vector is then selected randomly from the ecosystem as *X*_*j*_ to interact with the parasite vector, which replaces *X*_*i*_. X_j_ and Parasite_vector are then evaluated to find their fitness if *X*_*j*_ has the worst fitness compared with the Parasite_vector, the latter kills the former and replaces its position in the ecosystem otherwise, *X*_*j*_ will build immunity against the parasite_vector, which is then eliminated from the ecosystem. The Parasite_vector is obtained using Eq (7).


$$X_{parasite}=rand(0,1)\times (UB-LB)+LB$$ (7)

The clustering setup for the SOS algorithm used in this paper is like the one employed by [53], but it uses the common traditional based iterative process of assigning a maximum number of iterations to access the performance of the proposed algorithm instead of using the number of function evaluation. The standard algorithm steps of the SOS can be found in [49], while the modified version for automatic clustering is presented in Algorithm (1) below.

**Algorithm 1 Pseudocode for Standard SOS**

**Input:** ecosize: population size        SSUB: search space upper bound

      maxit: maximum number of iterations        SSLB: search space lower bound

      DD: problem dimension

      FF(X): fitness (objective) function

**Output:**      Xbest: the final global best solution for the population

**1:**      Generate initial population of organisms *X* = (*X*_1_, *X*_2_,**5:** …………..*X*_*ecosize*_)

**2:**      Evaluate the fitness of each organism

**3:**      Identify the initial population’s best solution X_best_

**4:**      **while *iteration*≤*maxIt***

**5:**          **for** i = *i* = 1 *to ecosize* **do**

**6:**          // Mutualism Phase //

**7:**          Randomly select index j (1 ≤ j ≤ ecosize; j ≠ i)

**8:**          *BF*_1_ = (1+*round*(*rand*(0,1)))

**9:**          *BF*_2_ = (1+*round*(*rand*(0,1)))

**10:**          $X_{mutual}=\left(\frac{X_{i}+X_{j}}{2}\right)$

**11:**          **for** *k* = 1 *to DD* **do**

**12:**            *X*_*inew*_ = *X*_*i*_+*rand*(0,1)*(*X*_*best*_−*BF*_1_*X_*mutual*_)

**13:**            *X*_*jnew*_ = *X*_*j*_+*rand*(0,1)*(*X*_*best*_−*BF*_2_*X_*mutual*_)

**14:**          **end for**

**15:**          if (*FF*(*X*_*inew*_)<*FF*(*X*_*i*_)

**16:**            *X*_*inew*_ = *X*_*i*_

**17:**          **end if**

**18:**          // Commensalism Phase//

**19:**          Randomly select index j (1 ≤ j ≤ ecosize; j ≠ i)

**20:**          **for** *k* = 1 *to DD* **do**

**21:**            *X*_*inew*_ = *X*_*i*_+*rand*(−1,1)*(*X*_*best*_−*X*_*i*_)

**22:**          **end for**

**23:**          **if** *FF*(*X*_*jnew*_)<*FF*(*X*_*j*_)

**24:**            *X*_*jnew*_ = *X*_*j*_

**25:**          **end if**

**26:**          //Parasitism Phase//

**27:**          Randomly select index j (1 ≤ j ≤ ecosize; j ≠ i)

**28:**          **for** *k* = 1 *to DD* **do**

**29:**          **if** *rand*(0,1)<*rand*(0,1)

**30:**            *X*_*parasite*_ = *X*_*i*_

**31:**    **else**

**32:**    *X*_*parasite*_ = *rand*(0,1)*(*SSUB*[*K*]−*SSLB*)+*SSLB*

**33:**    **end if**

**34:**    **end for**

**35:**    **if** *FF*(*X*_*parasite*_)<*FF*(*X*_*i*_)

**36:**    *X*_*i*_ = *X*_*parasite*_

**37:**    **end if**

**38:**    **end for**

**39:**    Update the best solution for the current population X_best_

**40:**    **end for**

**41:**    **end while**

### 3.2. K-Means algorithm

The K-means algorithm is a partitional clustering algorithm proposed by MacQueen in 1967 [44]. It is a simple clustering algorithm that is widely used for solving data clustering problems. K-means clustering algorithm has a linear time complexity (*O*(*n*) where *n* represents the number of data objects in the dataset. It has a high computational speed and can easily identify high density regions within a dataset. The k-means algorithm is listed as one of the top ten most used algorithms for data mining process [16], with wide acceptability for its simple implementation, low computational complexity, flexibility, and efficiency. The algorithm is made up of two separate phases. In the first phase, *k* number of data objects are randomly selected as cluster centres called centroids. The remaining data objects are then assigned to the closest cluster centre using the Euclidean distance metric to determine the distance between the object and the cluster centres. Once all data objects have been assigned, the average of the early formed clusters is then recalculated to determine the new centroid for the group. The iterative procedure is then repeated until the criterion function is minimum. K-means clustering requires user-specified parameter k as its input and generates k numbers of clusters. as its output

Given a dataset X containing n numeric objects such that *X*_*i*_ = (*x*_1_, *x*_2_,…..*x*_*n*_) and an integer number *k* representing the number of clusters in *X* with *k*< = *n*, the K-means algorithm partitions *X* into *k* clusters minimizing the within-cluster square errors. The mathematical formulation for the partitional clustering problem *F* for the K-means algorithm is as given in Eq (8) subject to the expression in Eq (9):

$$Minimize\;F(A,C)=\sum_{l=1}^{k}\sum_{i=1}^{n}a_{i,l}d(X_{i},C_{l})$$ (8)

$$subject\;to\;\sum_{l=1}^{k}a_{i,l}=1,\;1\leq i\leq n$$

$$such\;that\;a_{i,l}\in \{0,1\},\;1\leq i\leq n,\;1\leq l\leq k$$ (9)

where *A* is a partitional matrix of size *n*×*k* and ***C*** = *C*_1_, *C*_2_,…….,*C*_*k*_ represents a set of objects in the same cluster with *d* representing the square distance between two objects. The basic K-means algorithm pseudocode is presented in Algorithm 3 below.

**Algorithm 2 Pseudocode for Standard K-means**

**Input:**    Array {x_1_,x_2_,x_3_,…….x_n_}    // Dataset to be clusters

        k    // Number of required clusters

        CC{cc_1_,cc_2_,cc_3_,……cc_k_}    // Cluster centroids

**Output:**    A set of clusters

**1.**    // Initialize Parameters

**2:**    *X* = (*x*_1_,*x*_2_,*x*_3_,…….*x*_*n*_})

**3:**    *CC* = (*cc*_1_, *cc*_2_, *cc*_3_,……*cc*_*k*_})

**4:**    Repeat

**5:**        //Distance Calculations

**6:**        for *i* = 1 *to n* do

**7:**            for *j* = 1 *to k* do

**8:**                Compute Euclidean distance from a data object to all cluster

**9:**            end j

**10:**        //Data object assignment

**11:**          Add data objects to the closest cluster

**12:**        end i

**13:**        //Update cluster centroid

**14:**          Compute the new cluster centroid

**15:**    Until the difference between the cluster centroids of the two consecutive iterations remains the same

**16:**    End

### 3.3. Hybrid symbiotic organism search K-means optimization algorithm

The hybridization technique discussed in this paper aims to boost the classical K-means performance and extends its capability to solve automatic clustering problems by integrating it with the Symbiotic organism search algorithm. The hybridization strategy employed is like the one adopted in [63], where the strategy was used for dynamic clustering using binary Particle Swarm Optimization to generate the initial cluster centroid, and K-means were used to fine-tune the generated cluster centroids. However, in our study, the global exploration ability of the parameter-less SOS algorithm was combined with the exploitation ability of the simple K-means algorithm. The strategy balances the searching process for optimum cluster solution while ensuring non-convergence of K-means to a local minimum and simultaneously avoiding parameter tunning of other metaheuristic algorithms. In this study, there are two major sections in the SOSK-means algorithm. In the first part, the hybridized optimization algorithm commences the clustering process using the SOS algorithm. The optimum cluster centroids obtained during the three-phased SOS operations are then passed on to the K-means algorithm as the initial cluster centres.

During the initialization process of the SOS algorithm for clustering, *X* = (*X*_1_, *X*_2_, …………..*X*_*N*_) is set for *N* organisms to represent the initial population. Each organism *X*_1_ is a *k*×*m* dimensional vector with *k* representing the number of clusters and *m* representing the dimensions of the dataset *D*_*n*×*m*_. Each organism in the ecosystem is denoted as *X*_*i*_ = (x*1, x*2……….x*k). The minimum of each column in the dataset *D*_*n*×*m*_ is set as the lower bound x*j(*j* = 1,2,….*k*) representing one of the clustering centres, that is, a*j = *min* (*D*_1_, *D*_2_,………..*D*_*n*_) while the upper bound b*j = max (*D*_1_, *D*_2_,………..*D*_*m*_) is set as the maximum of each column in the dataset. The solution space for the clustering problem is bounded by the defined lower and upper bounds *a* and *b*, respectively. The organisms are uniformly and randomly distributed throughout the ecosystem, while the search space is limited to the solution space delineated by the defined lower and upper bounds. In solving the clustering problem, the *x*, which represents the *i*^*th*^ organism is obtained using the Eq (10) below:

$$X_{i}=rand(1,K\times m)*(b-a)+a$$ (10)

Where *rand*(1, *K*×*m*) represents a vector of uniformly distributed random numbers between 0 and 1. For the mutualism phase of the SOS, Eqs (4) and (5) are used to generate the new candidate organisms, while for commensalism and parasitism phases, Eqs (6) and (7) are adopted.

Algorithm 3 Pseudocode for Hybrid SOSK-means

**Input:**    ecosize: population size    SSUB: search space upper bound

maxit: maximum number of iterations    SSLB: search space lower bound

DD: problem dimension    FF(X): fitness (objective) function

**Output:**    Optimal Solution

**1**    Generate initial population of organisms *X* = (*X*_1_, *X*_2_ …………..*X*_*ecosize*_)

**2**    Evaluate the fitness of each organism

**3**    Identify the initial population’s best solution *X*_*best*_

**4**    **while** *iteration≤maxIt*

**5**        **for** *i* = 1 *to ecosize* **do**

**6**            // Mutualism Phase //

**7**            Randomly select index j (1≤*j*≤*ecosize*; *j*≠*i*)

**8**            *BF*_1_ = (1+*round*(*rand*(0,1)))

**9**            *BF*_2_ = (1+*round*(*rand*(0,1)))

**10**            $X_{mutual}=\left(\frac{X_{i}+X_{j}}{2}\right)$

**11**            **For** *k* = 1 *to DD* **do**

**12**                *X*_*inew*_ = *X*_*i*_+*rand*(0,1)*(*X*_*best*_−*BF*_1_*X_*mutual*_)

**13**                *X*_*jnew*_ = *X*_*j*_+*rand*(0,1)*(*X*_*best*_−*BF*_2_*X_*mutual*_)

**14**            **end for**

**15**            **if** (*FF*(*X*_*inew*_)<*FF*(*X*_*i*_)

**16**                *X*_*inew*_ = *X*_*i*_

**17**            **end if**

**18**            // Commensalim Phase//

**19**            Randomly select index j (1≤*j*≤*ecosize*; *j*≠*i*)

**20**            **for** *k* = 1 *to DD* **do**

**21**                *X*_*inew*_ = *X*_*i*_+*rand*(−1,1)*(*X*_*best*_−*X*_*i*_)

**22**            **end for**

**23**            **if** *FF*(*X*_*jnew*_)<*FF*(*X*_*j*_)

**24**                *X*_*jnew*_ = *X*_*j*_

**25**            **end if**

**26**            //Parasitism Phase//

**27**            Randomly select index j (1≤*j*≤*ecosize*; *j*≠*i*)

**28**            **for** *k* = 1 *to DD* **do**

**29**                **if** *rand*(0,1)<*rand*(0,1)

**30**                    *X*_*parasite*_ = *X*_*i*_

**31**                **else**

**32**                    *X*_*parasite*_ = *rand*(0,1)*(*SSUB*[*K*]−*SSLB*)+*SSLB*

**33**                **end if**

**34**            **end for**

**35**            **if** *FF*(*X*_*parasite*_)<*FF*(*X*_*i*_)

**36**                *X*_*i*_ = *X*_*parasite*_

**37**            **end if**

**38**            Update the best solution for the current population X_best_

**39**            //K-means Clustering Section//

**40**            Initialize the K-means with the position of the X_best_

**41**            Perform K-means clustering

**42**        **end for**

**43**        iteration = iteration + 1

**44**    **end while**

The algorithm starts with the ecosystem initialization process, where the number of organisms, and maximum iteration required are stated. The initial population of organisms of size *npop* are also generated. Next, the iterative procedure for the SOS algorithm is initiated. The fitness functions DB index or CS index are evaluated by evaluating each organism’s fitness in the initial population using the fitness function. The best fitness value is kept as the Xbest, representing the optimum solution for the initial population. The control is then passed on to the mutualism phase of the algorithm. During the mutualism phase of the algorithm, an organism X_j_ is randomly selected to interact with X_i_. The benefit factor for the two organisms is evaluated and used to generate their mutual benefit values. Two organisms, X_inew_ and X_jnew,_ are then generated using the mutual benefit values. The fitness function of the two new organisms, X_inew_ and X_jnew,_ are evaluated and compared with the X_i_ and X_j_ organisms. If X_inew_ and X_jnew_ organisms are more fitted, X_i_ and X_j_ organisms are replaced with X_inew_ and X_jnew_ and if otherwise, the X_i_ and X_j_ organisms will be retained while the new ones are discarded.

**Fig 1:**
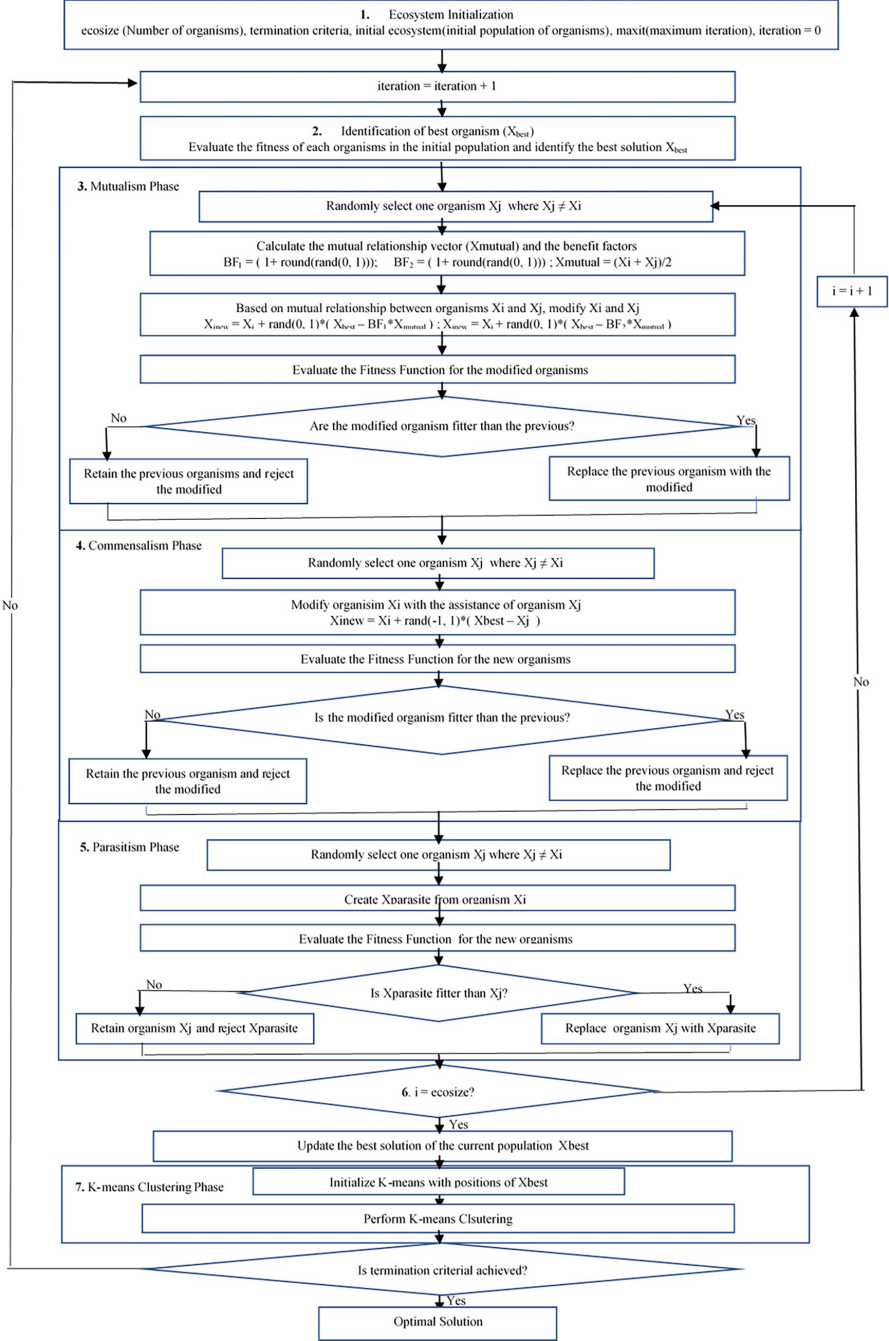
Proposed hybrid SOSK-means clustering algorithm.

The control is then passed on to the commensalism phase of the algorithm. At this level, an organism X_j_ is randomly selected, which is used in modifying organism X_i_ to generate a new organism X_inew_. The fitness function for the new organisms X_inew_ is then evaluated and compared with the X_i_. If X_inew_ organisms are more fitted than Xi organisms, Xi will be replaced with Xinew; otherwise, the X_i_ organisms will be retained while X_inew_ will be discarded. At this point, the parasitism phase takes over the control from the commensalism phase. As is the case with the initial two phases, an organism Xj is randomly selected and modified to be a new organism X_parasite_. The fitness function for the new organisms X_inew_ is then evaluated and compared with the X_i_. If X_inew_ organism is fitter than X_i_ organisms, X_i_ will be replaced with Xinew with Xi discarded and if otherwise, X_i_ will be retained while Xparasite is discarded. The procedure involving the three phases is performed on each organism in the dataset, and the X_best_ for the current population is updated. At the level, the output is fed into the classical K-means algorithm as input, supplying the value for k and the corresponding data points of the X_best_ obtained. The K-means clustering is then performed, and the result is given as the output for the iteration ith. The entire procedure is repeated until the maximum iteration is reached and the program terminates. The program flowchart showing the above procedure is presented in Fig 1 below:

### 3.4. Cluster validity indices

One of the fundamental parts of the clustering process is the validation of the results obtained from the clustering algorithms [90]. The cluster validation technique finds a set of clusters that best fits the natural partitions of datasets without prior knowledge of the class information regarding the dataset. It involves estimating how well a partition fits the underlying structure of the data. In this study, the Davies-Boulding index and the Compact Separated index are used to estimate the quality of the clustering results of the proposed SOSK-means algorithm. The two CVI are presented as an optimization problem where the objective function (the CVI) is required to be minimized. In each case, the lower the CVI value, the better the clustering result with better compactness and farther clusters separations. The CVIs are calculated as the fitness function for the SOSK-means clustering algorithm.

#### Davies-boulding index

The Davies-Boulding index (DB) evaluates the intra-cluster, that is, the average of all data points distance from the cluster centroid within a cluster and the inter-cluster distance between the centroids of two clusters to determine the quality of the clustering results produced by a clustering algorithm. The Davies-Boulding index is presented in Eq (11) below:

$$DB=\frac{1}{k}\sum_{i=1}^{k}\begin{array}{c} Max\\ i\neq j \end{array}\left\{\frac{d(x_{i})+d(x_{j})}{d(k_{i},k_{j})}\right\}$$ (11)

Where *k* is the number of clusters with *i*, *j* as the cluster labels. The *d*(*x*_*i*_) and *d*(*x*_*j*_) are the average of the distances of each data points to their respective cluster centroid within each cluster. The *d*(*k*_*i*_, *k*_*j*_) is the inter-cluster distance between cluster centroids of cluster *k*_*i*_ and cluster *k*_*j*_.

#### Compact-separated index

The Compact-Separated index (CS) uses the ratio of the sum of the within-cluster scatter to the between-cluster separation to measure the quality of a clustering result. Lower values of the CS reflect well-separated and more compacted clusters. It is reported as a better CVI in terms of the efficient handling of clusters with different densities, dimensions, and sizes. The Compact-Separated index is presented in Eq (12) below:

$$CS=\frac{\sum_{i=1}^{k}\left[\frac{1}{Q_{i}}\sum_{X_{i}\epsilon Q}{max}_{X_{j}\epsilon Q}\{V(X_{i},X_{j})\}\right]}{\sum_{i=1}^{k}\left[{min}_{j\epsilon k,j\neq i}\{V(x_{i},x_{j})\}\right]}$$ (12)

where the number of data points in cluster *C* is represented as |*D*| while the distance between the within-cluster scatter *X*_*i*_ and between-cluster separation *X*_*j*_ is represented as function *V*(*X*_*i*_, *X*_*j*_). The number of clusters in *Q* is given as *k*, and the distance of the data points d from their centroid is represented as *V*(*x*_*i*_, *x*_*j*_).

## 4. Experimentation

This section presents the description of the experimental configuration for evaluating the performance of the proposed hybrid SOSK-means for automatic data clustering and perimeter settings. The benchmark datasets used for validating SOSK-means’ performance alongside is also described. In the latter part of the section, the simulation results are presented with discussions on the result as well as their comparison with results from the literature.

### 4.1. Parameter setting and system configuration

The SOSK-means algorithm was programmed using MATLAB R2018b, while IBM SPSS Version 25 was used for the statistical analysis test to validate the statistically significant difference in the experimental results. The experiments were performed on a 3.60GHz Intel®Core™ i7-7700 processor with a memory size of 16GB running Windows 10 as the operating system. The simulation results of SOSK-means and results of existing algorithms published in the literature were compared to evaluate their performance. The algorithms include SOSTLBO [1], SOSFA [1], SOSPSO [1], SOSDE [1], DE [91], DCPSO [92]and GCUK [93]. Tables one contains the parameter settings for the SOSK-means, while Table 2 contains the setting for the other algorithms from the literature. In reporting the computed numerical solutions of the SOSK-means, the descriptive statistics employed include the best cost, the worst cost, the average cost, and the standard deviation. The report gives the clustering performance of the SOSK-means algorithm with reference to the defined objective functions using the DB index and CS index. The report also includes the average computational time the proposed hybrid algorithm spent to obtain the clustering solutions. The configuration of the parameter for SOS, K-means and SOSK-means is presented in Table 3.

**Table 2:** Initial parameter setting for classical SOS, classical K-means and proposed hybrid SOSK-means.

SOS		K-means		SOSK-means	
Parameter	Value	Parameter	Value	Parameter	Value
Max-It	200	k	As per dataset	Max-It	200
np	20	cc_1_….cc_k_	First k elements in dataset	np	20

**Table 3:** Initial parameter setting for the compared algorithms.

DCPSO		GCUK	
Algorithm’s Parameter	Assigned Value	Algorithm’s Parameter	Assigned Value
Popl_size	100	Popl_size	50
Inertial Weight	0.7200	Cross-over	0.800
c_1_, c_2_	1.4940	Mutation probability	0.0010
*K*_*max*_	20	*K*_*max*_	20
*K*_*min*_	2	*K*_*min*_	2

In total, eleven datasets, including both real-life and artificial datasets with two moons datasets, were used to evaluate the proposed algorithm’s performance and effectiveness compared to the other algorithms. However, not all the datasets used were included in the ones reported in the literature.

### 4.2. Clustering dataset

The eleven benchmark datasets used were obtained from the UCI Machine Learning Repository of the University of California. The summary of the eleven datasets used is given in Table 4, showing the data type, the dimension of the dataset, the number of data points and the number of clusters.

**Table 4:** Dataset characteristics.

Datasets	Dataset Features	Number of Clusters	Number of Objects	Dataset Type
Breast [58,94]	9	2	699	UCI
Compound [58,95]	2	6	399	Shape
Flame [58,96]	2	2	240	Shape
Glass [58,94]	9	7	214	UCI
Iris [58,94]	4	3	150	UCI
Jain [58,97]	2	2	373	Shape
Path-based [58,98]	2	3	300	Shape
Spiral [58,98]	2	2	312	Shape
Thyroid [58,94]	5	2	215	UCI
Two-moons [99]	2	2	10,000	-
Wine [58,94]	13	3	178	UCI
Yeast [58,94]	8	10	1,484	UCI

Each of the three individual algorithms (SOS, K-means and SOSK-means) was executed separately during the experimentation. The SOS and SOSK-means used an initially randomly generated population of size twenty-five over two hundred iterations. The regular procedure was adopted for the standard k-means algorithm with k given as the number of clusters for the corresponding dataset and the initial cluster centers generated randomly. However, in SOSK-means, the value for k and the corresponding optimum cluster centres generated by SOS was passed as an input parameter to the K-means phase. The algorithms were repeated forty independent times for all the datasets. Table 5 shows the numerical analysis of the validity measures on SOSK-means using DB and CS indexes on the twelve datasets. The best solution, worst solution, average solution and the standard deviation are denoted using the Best, Worst, Average and Std-Dev, respectively.

**Table 5:** SOSK-means results in over forty independent runs with DB and CS validity indices as the fitness function.

	DBIndex				CSIndex			
Dataset	Best	Worst	Average	Std Dev	Best	Worst	Average	Std Dev
**Breast**	0.8121	0.8121	0.8121	0.0000	**0.5996**	0.9574	0.7606	0.1217
**Compound**	**0.4974**	0.5158	0.5046	0.0044	0.5032	0.5918	0.5072	0.0155
**Flame**	0.7755	0.7787	0.7770	0.0008	**0.3846**	0.3846	0.3846	0.0000
**Glass**	0.3633	0.8159	0.7113	0.1217	**0.0608**	0.0608	0.0608	0.0000
**Iris**	0.5937	0.6744	0.6346	0.0188	**0.5367**	0.6444	0.5743	0.0237
**Jain**	**0.6490**	0.6535	0.6518	0.0009	0.6546	0.6546	0.6546	0.0000
**Pathbased**	0.6579	0.6740	0.6708	0.0031	**0.5961**	0.6894	0.6511	0.0120
**Spiral**	0.7350	0.7541	0.7437	0.0045	**0.6450**	0.6862	0.6812	0.0115
**Thyroid**	**0.5754**	0.6934	0.6321	0.0316	0.6409	0.6409	0.6409	0.0000
**Twomoons**	**0.6008**	0.6048	0.6032	0.0010	0.7176	0.7664	0.7498	0.0162
**Wine**	1.0045	1.0896	1.0460	0.0207	**0.6570**	0.8829	0.8422	0.0527
**Yeast**	0.4460	1.0819	0.8496	0.1588	**0.3897**	0.6303	0.5242	0.0437
**Average**	0.6426	0.7623	0.7197	0.0305	**0.5321**	0.6325	0.5860	0.0248

**Fig 2:**
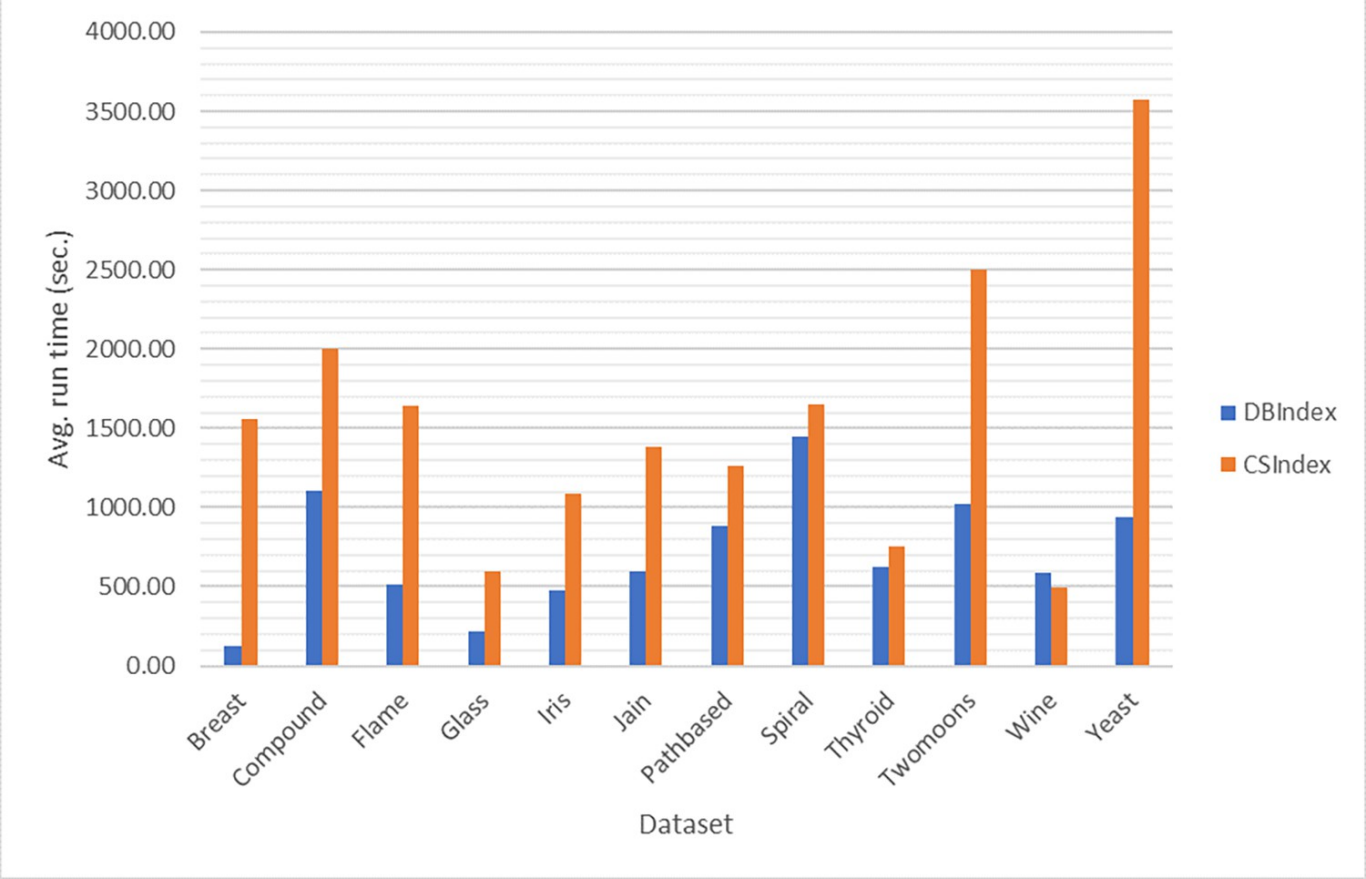
The mean run time achieved by SOSK-means on DB and CS measures over forty independent runs for the twelve datasets.

Fig 2 shows the average run time required by each of the data sets to obtain the optimal solution for by SOSK-means. The comparison of the simulated results for the three algorithms and those of the algorithms obtained from literature are presented respectively in Tables 6 and 7. For statistical validation of the significant difference between the different clustering results obtained from the various algorithms, Friedman test statistic and the Wilcoxon posthoc tests were conducted, and the results are presented in Tables 8 and 9, respectively.

**Table 6:** SOSK-means compared with SOS and K-means for forty replications.

Dataset	Algorithm	DBIndex		CSIndex	
		Mean Sol.	Std Dev	Mean Sol.	Std Dev
Breast	SOS	1.3520	0.2858	0.9946	0.2667
	Kmeans	0.8121	0.0000	1.1019	0.0000
	SOSKmeans	0.8121	0.0000	**0.7606**	0.1217
Compound	SOS	0.6924	0.1481	0.5670	0.1225
	Kmeans	0.9716	0.0748	1.2887	0.1486
	SOSKmeans	**0.5046**	0.0044	0.5072	0.0155
Flame	SOS	0.8234	0.0180	1.2707	0.1006
	Kmeans	1.2306	0.0059	1.5806	0.0263
	SOSKmeans	0.7770	0.0008	**0.3846**	0.0000
Glass	SOS	0.8164	0.1174	0.2200	0.2563
	Kmeans	1.2208	0.1570	1.4894	0.1904
	SOSKmeans	0.7113	0.1217	**0.0608**	0.0000
Iris	SOS	0.8602	0.1809	0.8585	0.1922
	Kmeans	0.9167	0.0033	1.2404	0.0092
	SOSKmeans	0.6346	0.0188	**0.5743**	0.0237
Jain	SOS	0.7007	0.0274	0.8196	0.0212
	Kmeans	0.8587	0.0001	1.0668	0.0003
	SOSKmeans	**0.6518**	0.0009	0.6546	0.0000
Pathbased	SOS	0.7578	0.0686	1.0021	0.1708
	Kmeans	0.7696	0.0066	0.9893	0.0086
	SOSKmeans	0.6708	0.0031	**0.6511**	0.0120
Spiral	SOS	0.8013	0.0447	1.0818	0.2107
	Kmeans	0.9589	0.0109	1.1896	0.0053
	SOSKmeans	0.7437	0.0045	**0.6812**	0.0115
Thyroid	SOS	1.0232	0.1479	0.6446	0.0238
	Kmeans	1.0298	0.2042	1.7863	0.3602
	SOSKmeans	**0.6321**	0.0316	0.6409	0.0000
Twomoons	SOS	0.6128	0.0179	0.7701	0.0281
	Kmeans	0.7948	0.0000	0.9385	0.0000
	SOSKmeans	**0.6032**	0.0010	0.7498	0.0162
Wine	SOS	1.1488	0.1394	1.1938	0.3318
	Kmeans	1.3053	0.0022	1.4425	0.0128
	SOSKmeans	1.0460	0.0207	**0.8422**	0.0527
Yeast	SOS	1.2144	0.2911	0.5594	0.2847
	Kmeans	1.7176	0.1875	2.6417	0.5950
	SOSKmeans	0.8496	0.1588	**0.5242**	0.0437

**Table 7:** SOSK-means results compared with results from existing algorithms in the literature.

Dataset	Algorithms	DBIndex		CSIndex	
		Mean	Std Dev	Mean	Std Dev
Breast	SOSKmeans	0.8121	0	0.7606	0.1217
	SOS	1.352	0.2858	0.9946	0.2667
	Kmeans	0.8121	0	1.1019	0
	SOSTLBO	0.8937	0.0384	-	-
	SOSFA	0.7644	0.0211	-	-
	SOSPSO	0.7128	0.1458	-	-
	SOSDE	1.1378	0.0947	-	-
	DE	0.5199	0.007	0.8984	0.381
	DCPSO	0.5754	0.073	0.4854	0.009
	GCUK	0.6328	0.002	0.6089	0.016
Glass	SOSKmeans	0.7113	0.1217	0.0608	0
	SOS	0.8164	0.1174	0.22	0.2563
	Kmeans	1.2208	0.157	1.4894	0.1904
	SOSTLBO	0.7832	0.0357	-	-
	SOSFA	0.6707	0.0459	-	-
	SOSPSO	0.6318	0.0418	-	-
	SOSDE	0.8444	0.0216	-	-
	DE	1.6673	0.004	0.7782	0.643
	DCPSO	1.5152	0.073	0.7361	0.671
	GCUK	1.8371	0.034	0.7282	2.003
Iris	SOSKmeans	0.6346	0.0188	0.5743	0.0237
	SOS	0.8602	0.1809	0.8585	0.1922
	Kmeans	0.9167	0.0033	1.2404	0.0092
	SOSTLBO	0.634	0.0182	-	-
	SOSFA	0.591	0.0075	-	-
	SOSPSO	0.5714	0.0038	-	-
	SOSDE	0.6916	0.0267	-	-
	DE	0.5822	0.067	0.7633	0.039
	DCPSO	0.6899	0.008	0.6899	0.008
	GCUK	0.7377	0.065	0.7377	0.65
Spiral	SOSKmeans	0.7437	0.0045	0.6812	0.0115
	SOS	0.8013	0.0447	1.0818	0.2107
	Kmeans	0.9589	0.0109	1.1896	0.0053
	SOSTLBO	0.7412	0.042	-	-
	SOSFA	0.7388	0.003	-	-
	SOSPSO	0.7332	0.0053	-	-
	SOSDE	0.7453	0.004	-	-
	DE	-	-	-	-
	DCPSO	-	-	-	-
	GCUK	-	-	-	-
Thyroid	SOSKmeans	0.6321	0.0316	0.6409	0
	SOS	1.0232	0.1479	0.6446	0.0238
	Kmeans	1.0298	0.2042	1.7863	0.3602
	SOSTLBO	0.6148	0.0234	-	-
	SOSFA	0.5313	0.0077	-	-
	SOSPSO	0.5021	0.0483	-	-
	SOSDE	0.7172	0.0532	-	-
	DE	-	-	-	-
	DCPSO	-	-	-	-
	GCUK	-	-	-	-
Wine	SOSKmeans	1.046	0.0207	0.8422	0.0527
	SOS	1.1488	0.1394	1.1938	0.3318
	Kmeans	1.3053	0.0022	1.4425	0.0128
	SOSTLBO	1.0413	0.0242	-	-
	SOSFA	0.9229	0.0189	-	-
	SOSPSO	0.8489	0.0741	-	-
	SOSDE	1.1108	0.0399	-	-
	DE	3.3923	0.092	1.7964	0.802
	DCPSO	4.3432	0.232	1.8721	0.232
	GCUK	5.3424	0.343	1.5842	0.343
Yeast	SOSKmeans	0.8496	0.1588	0.5242	0.0437
	SOS	1.2144	0.2911	0.5594	0.2847
	Kmeans	1.7176	0.1875	2.6417	0.595
	SOSTLBO	0.8954	0.0236	-	-
	SOSFA	0.7518	0.0346	-	-
	SOSPSO	0.7599	0.0666	-	-
	SOSDE	0.9869	0.0312	-	-
	DE	-	-	-	-
	DCPSO	-	-	-	-
	GCUK	-	-	-	-

**Table 8:** The Friedman means rank test results for the SOS, K-means and hybrid SOSK-means algorithms.

Dataset	DBIndex			CSIndex		
	SOS	Kmeans	SOSKmeans	SOS	Kmeans	SOSKmeans
Breast	3.00	**1.50**	**1.50**	2.25	2.45	**1.30**
Compound	1.95	2.95	**1.10**	1.65	3.00	**1.35**
Flame	2.00	3.00	**1.00**	2.00	3.00	**1.00**
Glass	1.73	3.00	**1.27**	1.84	3.00	**1.16**
Iris	2.35	2.48	**1.18**	1.86	3.00	**1.14**
Jain	2.00	3.00	**1.00**	2.00	3.00	**1.00**
Pathbased	2.30	2.45	**1.25**	2.66	2.23	**1.11**
Spiral	1.83	3.00	**1.18**	2.29	2.58	**1.14**
Thyroid	2.40	2.53	**1.08**	1.51	3.00	**1.49**
Twomoons	1.66	3.00	**1.34**	1.73	3.00	**1.27**
Wine	2.03	2.70	**1.27**	2.14	2.63	**1.24**
Yeast	2.00	2.88	**1.13**	1.23	3.00	1.78

**Table 9:** Wilcoxon rank-sum test for equal medians showing corresponding *p*-values.

Dataset	DBIndex			CSIndex		
	SOS vs Kmeans	SOSKmeans vs SOS	SOSKmeans vs Kmeans	SOS vs Kmeans	SOSKmeans vs SOS	SOSKmeans vs Kmeans
Breast	0.000	0.000	1.000	0.034	0.000	0.000
Compound	0.000	0.000	0.000	0.000	0.006	0.000
Flame	0.000	0.000	0.000	0.000	0.000	0.000
Glass	0.000	0.002	0.000	0.000	0.000	0.000
Iris	0.147	0.000	0.000	0.000	0.000	0.000
Jain	0.000	0.000	0.000	0.000	0.000	0.000
Pathbased	0.301	0.000	0.000	0.053	0.000	0.000
Spiral	0.000	0.000	0.000	0.044	0.000	0.000
Thyroid	0.554	0.000	0.000	0.000	0.317	0.000
Twomoons	0.000	0.009	0.000	0.000	0.000	0.000
Wine	0.000	0.000	0.000	0.002	0.000	0.000
Yeast	0.000	0.000	0.000	0.000	0.021	0.000

In the comparison of the performance of SOSK-means with those obtained from other hybridized metaheuristics algorithms, the focus was on the clustering solution quality obtained from the DB and CS validity indices which were used as the fitness function, as well as the computation time is taken by the two validity indices to get the solution for the individual dataset. The results are presented as four decimal place values with an emphasis on datasets where SOSK-means performed better than the other algorithms. Such results are presented in bold font.

### 4.2. Results and discussion

It can be seen from the summarised result of Tables 5 and 6 that the proposed SOSK-means algorithm performed efficiently in solving automatic data clustering problems. Compared with the non-hybridized clustering algorithm SOS and K-means, the SOSK-means exhibited superior performance in some datasets. A comparison of the results obtained from forty independent runs for the DB and CS validity measures is shown in Table 4. For a fair comparison, a uniform initial population size of 25 was used for both SOS and SOSK-means. The performance of the proposed algorithm SOSK-means on the two cluster validity indices for all the datasets is shown in Table 4. The DB and CS as the objective functions for the proposed algorithms are cases of minimization problems with the least values indicating the best results. In eight of the datasets, better results were obtained by the CS-index compared with the DS-index, which recorded better results in Compound, Jain, Thyroids and Two-moons datasets.

From the performance results obtained for the two CVIs, it can be deduced that CS-index has a better solution with higher partitioning ability compared with the DB-index. The DB measure returned a lower computational time than the CS measure for most of the datasets. As stated earlier, the SOSK-means has its best optimal result for Compound, Jain, Thyroids and Two-moons datasets on the DB measure, while the best optimal result of the CS measure is recorded for the Breast, Flame, Iris, Path-based, Spiral, Wine, and Yeast datasets. DB measure has its worst result on the Wine dataset while the worst result for CS measure is recorded for Breast dataset.

The computational time for the two objective functions is presented in Fig 2, showing the average execution run time of the proposed algorithm for all the datasets for the forty independent runs. The CS-index has poor computational times compared with the DB-index in almost all the datasets. SOSK-means recorded a very high average runtime for the Yeast dataset CS index. Except for Spiral, Thyroid and Wine datasets, the average runtime recorded by CS-index is much higher than the DB-index for the remaining datasets.

The summarised results for the classical SOS, K-means and the SOSK-means algorithm are shown in Table 5. The results for the three algorithms using the mean solution and the standard deviation from the experiments involving forty independent runs are compared. For all the datasets, the SOSK-means algorithm performs better than the individual classical algorithms on the two CVIs. The proposed SOSK-means algorithm outperforms the standard K-means algorithms in all the datasets except the Breast dataset, where they have the same result on the DB-index. SOSK-means records the best means solution for Breast, Flame, Glass, Iris, Pathbased, Spiral, Wine and Yeast under CS-index and the best means solution for Compound, Jain, Thyroid and Twomoons under the DB-index. The overall results show that the SOSK-means performs better than the individual classical SOS and K-means algorithms in terms of solution quality.

A comparison of the performance of proposed SOSK-means with results of other clustering algorithms from literature is presented in Table 6. The performance of the individual algorithms, when executed on seven datasets, namely Breast, Glass, Iris, Spiral, Thyroid, Wine and Yeast, was used to measure their competitiveness. The analysis of the BD and CS measure performances is presented with the following observations from the analysis table.

Breast Cancer Wisconsin (Original) dataset: Table 6 shows that the result for SOSK-means and standard K-means are identical with the smallest Std-Dev values on the DB measure. However, the classical DE recorded the optimum mean value outperforming the proposed SOSK-means algorithm, with the GCUK having the minimum standard deviation under the DB measure. The DCPSO recorded the optimum mean value and the best standard deviation on the CS measures.

Glass dataset: The proposed SOSK-means algorithm obtained the smallest values for the computed average and standard deviation on the CS measure, while SOSPSO has the best mean value under the DB measure with the smallest standard deviation recorded under the classical DE.

Iris dataset: On the CS measure, the proposed SOSK-means algorithm outperformed all the other algorithms recorded with the lowest mean values, but DCPSO recorded the smallest variation on the two validity measures. On the other hand, SOSPSO outperformed the proposed algorithm on the DB measures outperforming all the listed algorithms.

Spiral dataset: On the CS measure, the proposed algorithm outperformed two of the other competing algorithms, while others were not available in the literature. However, for the DB measure, SOSPSO recorded the minimum mean value for the Spiral dataset, while SOSDE and the proposed algorithm have very close values for the mean and standard deviation. The proposed SOSK-means had a better mean value while SOSDE had better variation.

Thyroid dataset: As is the case with the Spiral dataset, on the CS measure, the proposed algorithm could only be compared with two competing algorithms, and the result showed its superior performance. The SOSPSO recorded the best mean value on the CS measure, with SOSFA recording the best variation. However, the proposed algorithm has a better variation compared with SOSPSO

Wine dataset: The proposed algorithm outperformed all the competing algorithms on the CS measure, with the classical K-means having the smallest variation on both measures. On the other hand, SOSPSO has the smallest mean value but with a higher DB measure variation than the proposed algorithm. The standard K-means algorithm recorded the lowest variation of Std-Dev on both measures.

Yeast dataset: For this dataset, the results for seven of the competing algorithm under the CS measures were not available. However, the proposed algorithm outperformed the available ones. Under the DB measure, the SOSFA has the optimum mean value, while the SOSTLBO has the least value for standard deviation.

In order to statistically validate the presented results, Friedman’s statistical test was conducted for further justification of the performance of the SOSK-means algorithm. The Friedman mean-rank test is a non-parametric test like the ANOVA. Any significant difference in the behavioural pattern of two or more algorithms can be established using Friedman’s test. The test was carried out on the standard SOS, standard K-means, and the proposed SOSK-means algorithms. The Friedman’s statistical test result is presented in Table 7. The proposed SOSK-means algorithm ranked better in all the datasets on the DB measure, while it recorded a better ranking in all but one, namely the Yeast dataset on the CS measure. On both measures, the proposed SOSK-means algorithm ranked better than the two classical algorithms SOS and K-means in almost all the datasets except in the Breast dataset, where it formed a tie with the standard algorithm and in the Yeast dataset, where SOS has a better rank than the proposed algorithm. From the Friedman’s ranking recorded, the SOSK-means can be concluded to be a strong, efficient, and effective algorithm with better performance than the two classical algorithms in handling automatic data clustering analysis. The proposed SOSK-means algorithm ranked better on the two CVI measures in eleven (11) datasets out of the twelve (12) datasets used.

SOSK-means has the same mean rank as the standard K-means algorithm for the Breast dataset on the DB measure with a better rank value than SOS. Table 7 shows the Friedman mean ranking, with SOSK-means values written in bold, where it ranked better than the other competing classical algorithms. The statistical result presented indicates that the SOSK-means algorithm ranked better than the SOS and K-means.

The Wilcoxon rank-sum test was performed as a post hoc test to compare the control method and other algorithms. Performing a post hoc test on the Friedman statistical test helps us avoid using it as the only basis for our statistical judgement. The post hoc test was conducted among SOS, K-means and SOSK-means. It established a statistical significance between the pairwise groups consisting of SOS and K-means, SOSK-means and SOS, SOSK-means and K-means. The computed *p* values showing the statistical significance between the set of the algorithm using the Wilcoxon rank-sum test for the equal median are presented in Table 8.

The statistical significance between the set of the algorithms is averagely the same on both measures for most of the datasets with values less than 0.05 significant value. The 0.05 represents a 5% significant level for accepting the null value. With more values less than 0.05, it establishes that the values are samples from a continuous distribution with equal medians against the null hypothesis that they are not. This gives convincing evidence that statistically establishes the significance of the results of the hybrid SOSK-means algorithm.

**Fig 3:**
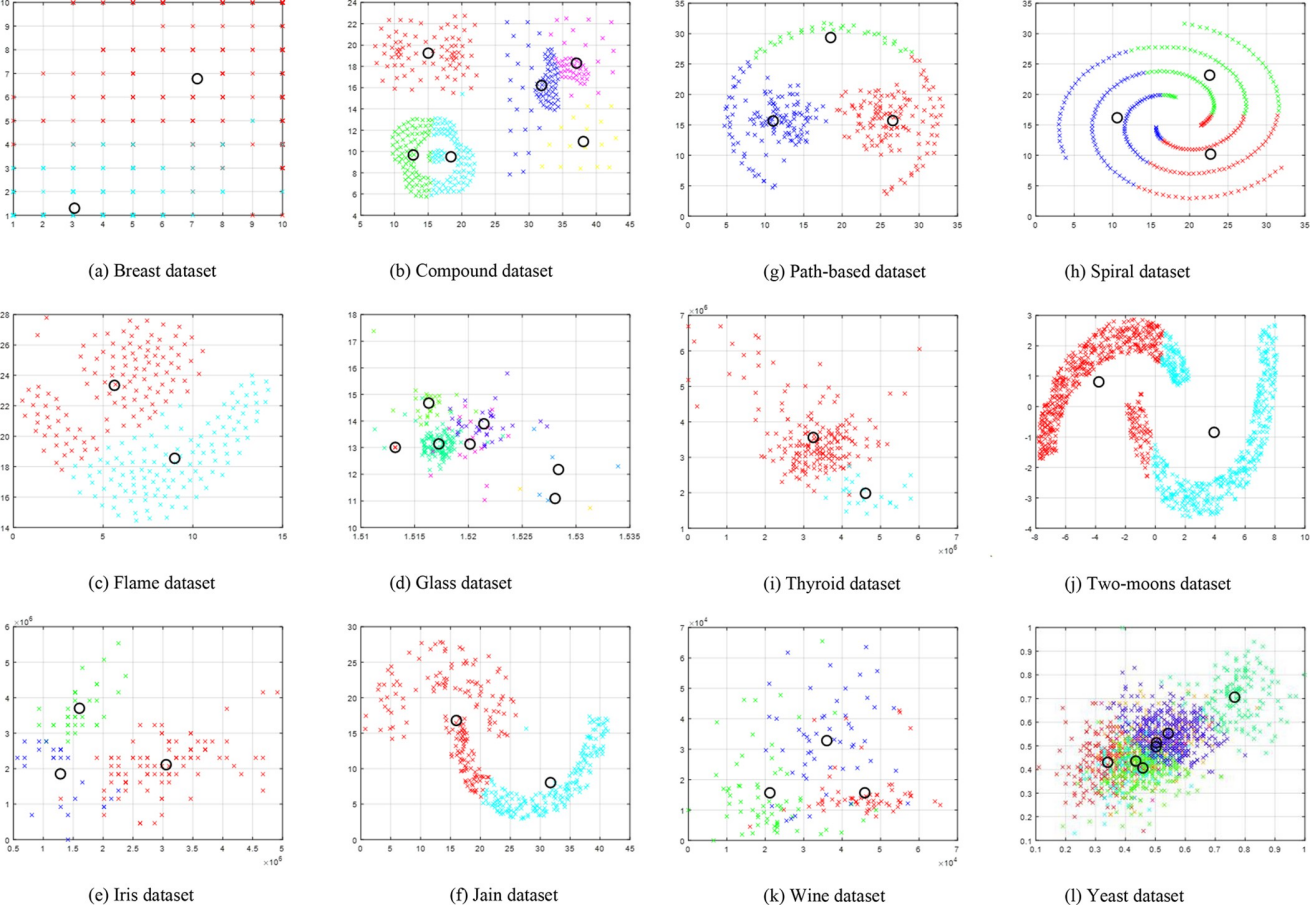
Clustering illustration of hybrid SOSK-means for the listed datasets using DB-Index.

**Fig 4:**
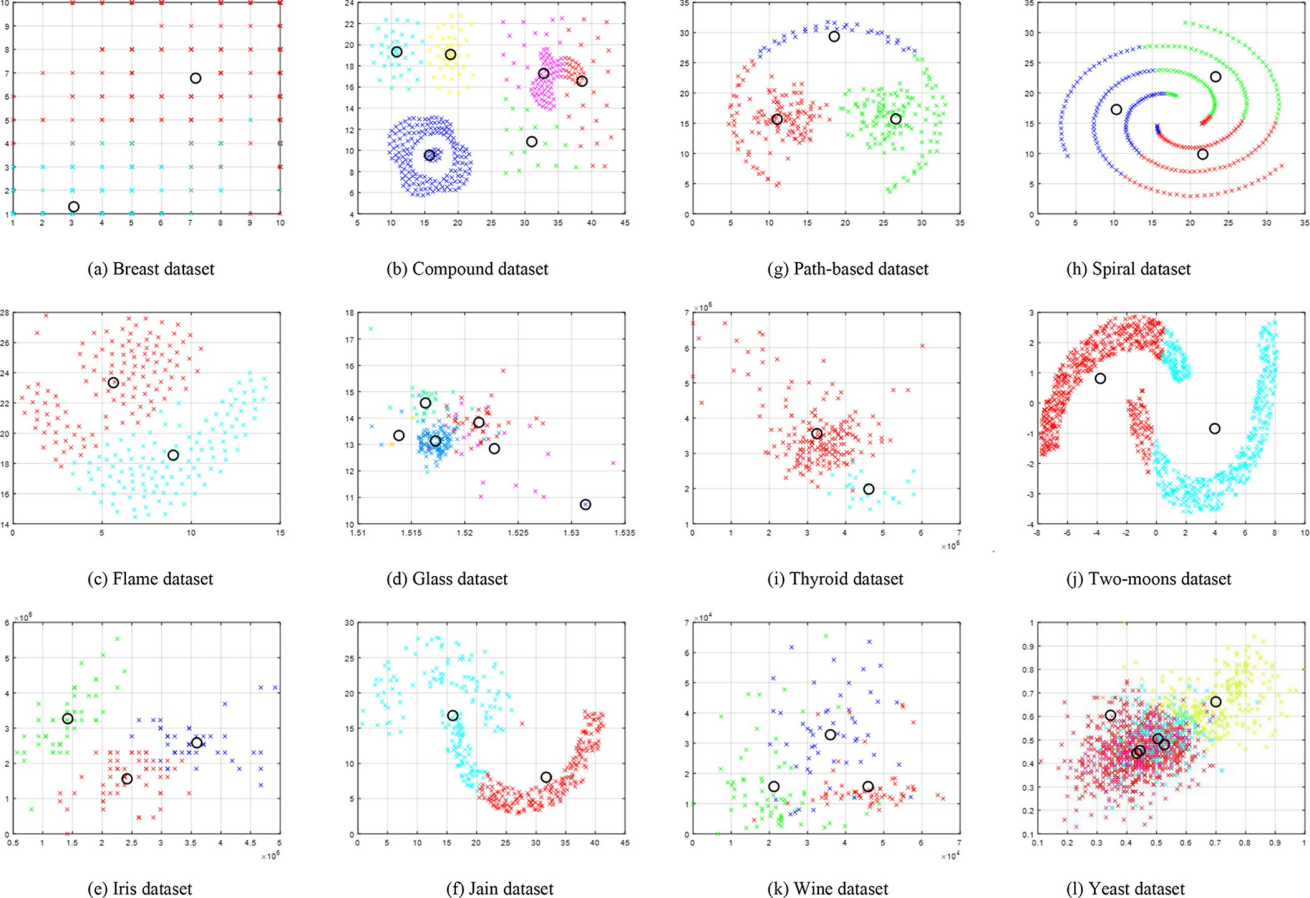
Clustering illustration of hybrid SOSK-means for the listed datasets using CS-Index.

Figs 3 and 4 show the individual clustering samples for the twelve datasets for the proposed hybrid SOSK-means on DB and CS measures. In Fig 3 (A) (Breast dataset), the dataset has a clear division into two classes, coloured red and blue, with the red class having a single outlier while the blue class has six outliers near its edges. Six distinct classes can be observed for the Compound dataset, with just a single outlier of the blue found in the red class. The flame dataset has a clear division into two distinct classes, the same as the Jain dataset and the two-moons dataset, with no outlier observed. The Glass dataset has five classes that can be distinctly identified and two interweaved (the pink and deep blue coloured classes). The Iris, Path-based, Thyroid and Spiral datasets are well demarcated into their various clusters. A few outliers can be spotted in the Iris and Thyroid datasets between the blue and red classes. The three classes in the Wine dataset can be seen though few data objects are spotted as outliers in the three classes. Seven classes were spotted in the Yeast dataset, with several overlapping data objects among the classes.

## 5. Conclusion and future research direction

This paper proposed and implemented an improvement to the classical K-means algorithm, a hybrid SOSK-means combining SOS with K-means for solving the automatic clustering problem. It addressed the classical K-means algorithm problems of given cluster numbers as input parameters and randomly selecting the initial cluster centers. The issue of local optimal convergence was also taken care of. SOSK-means automatically determines the optimal number of clusters in real-time, even for datasets with high dimensions. It combined the advantages of the SOS algorithm credited with its parameter-less attributes and the excellent local exploration of the K-means algorithm with implementation simplicity. The simulation results clearly demonstrate the superior performance of hybrid SOSK-means over the classical SOS and K-means. It also outperformed some of the competing metaheuristic algorithms. SOSK-means performance in finding the solution to the automatic clustering problem was statistically confirmed from its performances over most of the benchmark datasets used in the experimentation as reported from the Friedman rank test and the post hoc Wilcoxon rank-sum test for equal medians. The demarcated clustering results show the capability of the hybrid SOSK-means to achieve an optimal cluster number and improved convergence speed with better clustering solutions. The Compact Separated clustering validity index was a better and more effective clustering metric for the proposed hybrid SOSK-means algorithm, with a higher run time than the Davies Bouldin index. In terms of cohesion and compactness, the CSI reported better cluster solutions. Although the hybrid SOSK-means algorithm was able to resolve the initialisation problem of the traditional K-means algorithm, it is observed that the computational time required in the K-means phase is still proportional to the size of the dataset. Also, some of the hybridized algorithms involving two metaheuristics algorithms outperformed SOSK-means. This established the excellent performance of metaheuristic algorithms when finding solutions to clustering problems.

For future research directions, improved variants of K-means can be combined with SOS to reduce the local search time spent by the classical K-means. Also, improved versions of SOS can be introduced to enhance the hybrid algorithm’s performance further. For better comparison, other metaheuristics algorithms hybridized with K-means can be executed using the same dataset to show the proposed algorithm’s performance effectively. The performance of other clustering validity indexes on the proposed hybrid SOSK-means can also be explored. Having established the fact that the proposed hybrid SOSK-means algorithm is efficient in handling automatic clustering, it can be applied to solve different real-world problems in other related fields.
